# Defect-Mask2Former: An Improved Semantic Segmentation Model for Precise Small-Sized Defect Detection on Large-Sized Timbers

**DOI:** 10.3390/s26072254

**Published:** 2026-04-06

**Authors:** Mingming Qin, Hongxu Li, Yuxiang Huang, Xingyu Tong, Zhihong Liang

**Affiliations:** 1 College of Big Data and Intelligent Engineering, Southwest Forestry University, Kunming 650224, China; swfuqmm@swfu.edu.cn (M.Q.); yxhuang@swfu.edu.cn (Y.H.); tongxingyu@swfu.edu.cn (X.T.); 2 College of Materials and Chemical Engineering, Southwest Forestry University, Kunming 650224, China; 13085315910@163.com

**Keywords:** wood defect segmentation, small-sized defects, attention-guided pyramid enhancement, defect boundary calibration, glued laminated timber grading

## Abstract

The precise segmentation of small-sized defects on wood surfaces is critical for the quality grading of glued laminated timber (GLT). Existing semantic segmentation models face core bottlenecks in this context: high miss rates, blurred boundary localization, and excessive size measurement errors. To address these issues, this paper proposes an improved Defect-Mask2Former model that integrates an Attention-Guided Pyramid Enhancement (AGPE) module and a Defect Boundary Calibration and Correction (DBCC) module. Through synergistic optimization, the model achieved pixel-level precise segmentation. To support model training and validation, a custom image acquisition device was designed, and the PlankDefSeg dataset was constructed, comprising 3500 pixel-level annotated images covering five defect types across six industrial wood species. Experimental results demonstrate that on the PlankDefSeg dataset, Defect-Mask2Former achieved a mean Intersection over Union (mIoU) of 85.34% for small-sized defects, a 17.84% improvement over the baseline Mask2Former. The miss rate was reduced from 20.78% to 5.83%, and the size measurement error was only 2.86%, strictly meeting the ≤3% accuracy requirement of the GB/T26899-2022 standard. The model achieved an inference speed of 27.6 FPS, satisfying real-time detection needs. By integrating the model into the GLT grading workflow, a grading accuracy of 94.3% was achieved, and the processing time per timber was reduced from 30 s to 1.5 s, a 20-fold efficiency improvement. This study provides reliable technical support for intelligent GLT quality grading and offers a reference solution for other industrial surface defect segmentation tasks.

## 1. Introduction

Wood, as a renewable natural material, is widely used in fields such as building structures, furniture manufacturing, and interior decoration [1]. Glued Laminated Timber (GLT), formed by bonding together multiple layers of sawn timber to create large-scale structural components [2], effectively overcomes the limitations of natural wood’s size constraints and discrete mechanical properties. It has become a core load-bearing material in modern timber structures [3]. However, surface defects such as knots, cracks, and mineral streaks, which occur during wood growth and processing, can significantly reduce key mechanical properties like bending strength and modulus of elasticity of the GLT, directly impacting its quality grade and safety for engineering applications [4]. Therefore, precise detection and segmentation of wood surface defects is a core prerequisite for GLT quality grading.

According to the national standard GB/T26899-2022, “Structural glued laminated timber” [5], the core criteria for GLT quality grading include the diameter and area of individual defects, the concentrated knot diameter ratio (the ratio of defect diameter to board width), and the number and density of defects per unit area. All these indicators are based on pixel-level precise segmentation results of the defects, rather than merely relying on rectangular bounding boxes output by detection stages. Especially for small-sized defects (diameter < 3 mm), bounding box regression suffers from significant localization errors, making it difficult to accurately reflect the true size and morphology of the defects [6]. In GLT production practice, defects are categorized into three types by size: large defects (diameter ≥ 10 mm), medium defects (3 mm ≤ diameter < 10 mm), and small-sized defects (diameter < 3 mm) [7]. Small-sized defects mainly include fine cracks (width < 2 mm), small knots (diameter < 3 mm), and fine mineral streaks (width < 3 mm). Although individually small, when their density exceeds 3 per 100 cm^2^, they can significantly lower the GLT grade [8]. Existing detection methods have achieved a mean Average Precision (mAP) of over 85% for large-sized wood defects, mainly including one-stage detectors such as YOLOv3, YOLOv5, and SSD [9,10,11], as well as two-stage detectors such as Faster R-CNN [10]. However, the detection accuracy for small-sized defects with diameter < 3 mm is only about 70–75% [9], which becomes the main bottleneck restricting the overall performance of the grading system.

Semantic segmentation technology, by classifying each pixel in an image, can output the precise boundary contours of defects, providing a basis for defect size quantification. In recent years, semantic segmentation models have developed rapidly: the Fully Convolutional Network (FCN) [12] first introduced a fully convolutional structure to semantic segmentation, overcoming the size limitations of traditional classification models. The encoder–decoder architecture and skip connections proposed by U-Net [13] have become classic paradigms in medical and industrial defect segmentation. The DeepLab series [14] expands the receptive field using atrous convolutions, effectively capturing multi-scale contextual information. Transformer-based segmentation models, with their global modeling capabilities, have become a current research hotspot: SegFormer [15] employs a lightweight Transformer encoder and hierarchical feature fusion strategy, achieving excellent performance on multiple benchmark datasets. Mask2Former [16], with its mask classification paradigm, unifies semantic and instance segmentation. Unlike traditional pixel-wise classification methods (e.g., FCN [12]), Mask2Former formulates segmentation as a set of binary mask classifications. Its masked attention mechanism restricts each query’s focus to its corresponding mask region, and the Transformer architecture provides superior global modeling capability, leading to significantly better segmentation accuracy in general scenarios than traditional models. However, most of these general semantic segmentation models are designed for general scenes (e.g., medical images, urban scenes) and lack targeted optimization for wood small-sized defects. Specifically, they fail to address the core issues of weak feature response of small defects, severe interference from wood growth ring textures, and inaccurate boundary localization required for industrial measurement, leading to low segmentation accuracy and high miss rates in wood defect detection [10,11].

To address the issue of weak feature responses for small-sized objects, researchers have proposed various feature enhancement strategies. The Feature Pyramid Network (FPN) proposed by Lin et al. [17] constructs a multi-scale feature pyramid through top-down feature propagation and lateral connections, increasing the AP for small object detection by about 8 percentage points on the COCO dataset. The Path Aggregation Network (PANet) by Liu et al. [18] adds a bottom-up path aggregation on top of FPN, achieving bidirectional fusion of multi-scale features. The High-Resolution Network (HRNet) proposed by Wang et al. [19] maintains high-resolution feature representations throughout the network, effectively avoiding the loss of spatial information for small objects. In boundary optimization, the boundary-aware loss function proposed by Ke et al. [20] guides the network to focus on boundary pixels by increasing the gradient weight in boundary regions, significantly improving the IoU of segmentation boundaries. OCRNet by Yuan et al. [21] enhanced feature response in boundary regions through object-contextual representations. Li et al. [22] proposed the Semantic Flow Network (SFNet), which optimizes boundary localization accuracy through feature alignment. In the application domain of industrial surface defect segmentation, Tian et al. [23] systematically reviewed key technologies for small-sized industrial defect detection and segmentation, summarizing three core strategies: multi-scale feature fusion, high-resolution representation, and data augmentation. Tabernik et al. [24] proposed a segmentation-based method for industrial surface defect detection, transforming the detection task into a pixel-set segmentation task, achieving an AP of 97.2% in steel surface defect detection. Zhang et al. [25] designed a high-resolution representation network for fine-crack segmentation, achieving an F1-score of 86.2% for cracks with a width < 2 mm.

To address the aforementioned challenges, this paper proposes an improved Defect-Mask2Former model for wood small-sized defect segmentation, using Mask2Former as the baseline. Through the synergistic optimization of the AGPE and DBCC modules, precise segmentation of small-sized defects was achieved. To provide reliable location prior information for small-sized defects, we adopt the EFCW-YOLO model [26], a lightweight wood defect detection model proposed in our previous work, which achieves an mAP50 of 90.26% for small-sized defects and provides high-confidence candidate boxes for subsequent segmentation. The core contributions of this paper are as follows:

(1) We propose an original Attention-Guided Pyramid Enhancement (AGPE) module: This module integrates location prior information from an upstream detector. Through a three-stage mechanism of “feature purification–cross-scale enhancement–small-sized anchoring”, it specifically suppressed wood texture interference and enhanced feature responses for small-sized defects, reducing the small-sized defect miss rate from 20.78% to 5.83%.

(2) We design a Defect Boundary Calibration and Correction (DBCC) module: This module features a dual-branch parallel architecture and a boundary-aware loss function. It optimizes boundary localization specifically to meet the ≤3% size measurement accuracy requirement of the GB/T26899-2022 standard, reducing the diameter measurement error from 8.31% to 2.86% and achieving a deep integration of segmentation results with industrial grading needs.

(3) We construct a dedicated dataset for wood small-sized defects, PlankDefSeg: A custom image acquisition device was designed to build a dataset of 3500 pixel-level annotated images covering five types of small-sized defects across 6 industrial wood species. Three targeted data augmentation strategies are also proposed to address the issues of sparse samples and class imbalance for small-sized defects.

(4) We realize an end-to-end technical closed-loop for GLT grading: The segmentation results of Defect-Mask2Former are integrated into the GLT grading process, forming a standardized “segmentation–measurement–grading” pipeline. The system achieved a grading accuracy of 94.3%, and its efficiency was 20 times higher than manual grading, providing a complete solution for industrial implementation.

## 2. Results

### 2.1. Training Process Analysis

**Figure 1:**
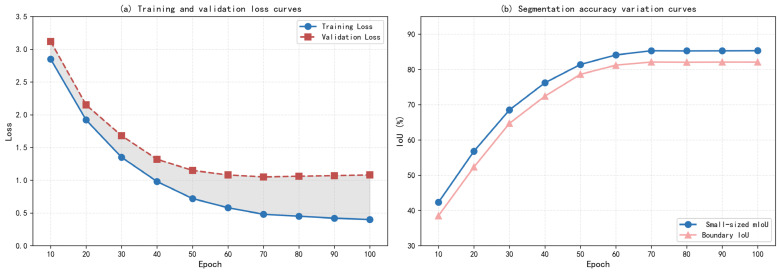
Model training process curves. (**a**) Training and validation loss curves; (**b**) Small-sized mIoU and boundary IoU variation curves.

Figure 1 showed the training process curves of the Defect-Mask2Former model. Table 1 presented the loss and performance metrics at key epochs during training. The model achieved optimal performance at the 70th epoch, after which training was stopped. The overall training process was stable, with no obvious overfitting.

From the training process curves in Figure 1 and Table 1, the following conclusions could be drawn:

(1) Fast Model Convergence: In the first 30 epochs, the training loss rapidly decreased from 2.85 to 1.35, a drop of 52.6%. The small-sized mIoU increased from 42.30% to 68.50%, a gain of 26.2 percentage points, indicating that the model could quickly learn features of wood small-sized defects.
(2) Epochs 30–50 as Fine-Tuning Period: During this phase, the loss decreased more slowly. The small-sized mIoU steadily improved from 68.50% to 81.40%, and the boundary IoU increased from 64.70% to 78.60%. The model began to focus on fine-grained learning of defect boundaries.
(3) Convergence after Epoch 50: After 50 epochs, the loss remained largely stable. The small-sized mIoU and boundary IoU slowly increased, reaching optimal values at epoch 70 (small-sized mIoU = 85.30%, boundary IoU = 82.10%). Thereafter, slight fluctuations due to mild overfitting occurred, but overall performance remained stable.
(4) Strong Model Generalization: The gap between training and validation loss remained consistently small. At optimal performance, the validation loss (1.05) was about 2.19 times the training loss (0.48), indicating no significant overfitting and strong generalization ability, which was attributable to the targeted data augmentation strategies and the lightweight model design.
(5) The generalization gap between training and validation losses at optimal performance was 0.57, and the overfitting coefficient was 1.19. The validation loss remained stable throughout training without a subsequent increase, indicating no significant overfitting. This is attributable to the data augmentation strategies, lightweight module design, and early stopping mechanism.

Under the established experimental environment, the training time per epoch for the Defect-Mask2Former model was about 12 min. Training to optimal performance (70 epochs) took approximately 14 h, demonstrating good training efficiency for subsequent model tuning and improvement, which was consistent with findings in efficient training strategies.

### 2.2. Baseline Model Comparison

**Figure 2:**
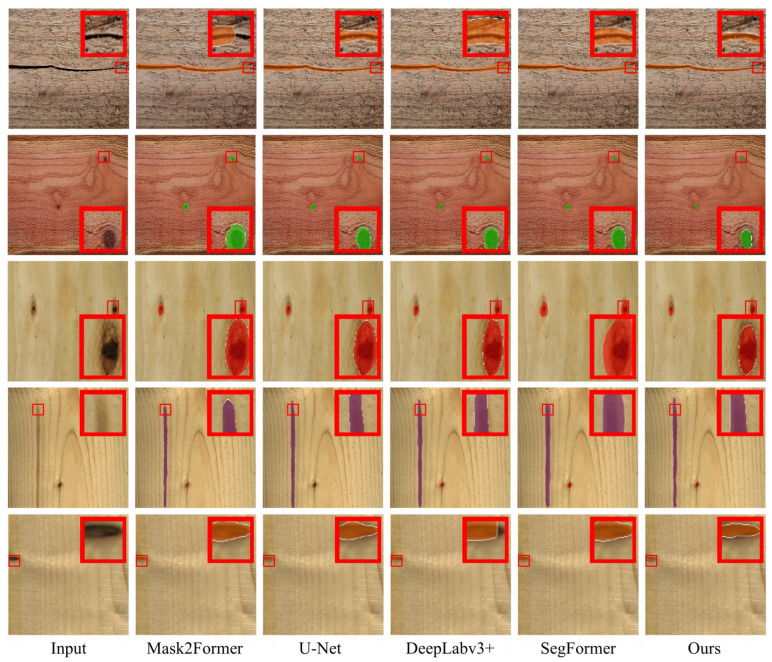
Performance comparison of different models on the PlankDefSeg test set. Note: From left to right are the segmentation results of the original image, Mask2Former, U-Net, DeepLabv3+, SegFormer, and our Defect-Mask2Former. The smaller red boxes mark the segmented defect regions, while the larger red boxes display the zoomed-in views of these regions to facilitate detailed observation of the model segmentation performance. Different colors are used to distinguish and label different types of wood defects in the segmentation results.

To validate the overall performance of the Defect-Mask2Former model, it was compared with four mainstream semantic segmentation models on the PlankDefSeg test set. These models were selected based on their representativeness (covering encoder–decoder, atrous convolution, and Transformer-based architectures), state-of-the-art performance, and applicability in industrial defect segmentation. All models were trained from scratch with the same hyperparameters to ensure fairness. Table 2 presented the core evaluation metrics for each model. The segmentation results of the baseline models were shown in Figure 2.

From the comparison results in Figure 2, it could be seen that the proposed Defect-Mask2Former model achieved the best performance across all evaluation metrics, comprehensively surpassing existing mainstream semantic and instance segmentation models. The detailed analysis was as follows:

Segmentation Accuracy Advantage: Defect-Mask2Former achieved a small-sized mIoU of 85.34%, a 10.09 percentage point improvement over SegFormer (75.25%). The core reason was the location prior guidance and cross-scale feature enhancement of the AGPE module, which solved the problem of weak feature response for small defects. The boundary IoU reached 82.11%, a 17.82 percentage point improvement over Mask2Former, demonstrating the precise calibration effect of the DBCC module on boundary localization.

Quantification Accuracy Met Industrial Needs: The size measurement error of 2.86% strictly met the ≤3% requirement of the GB/T26899-2022 national standard. In contrast, the measurement errors of comparison models all exceeded 6% (U-Net reached 10.38%), making them unable to support the calculation of quantitative indicators for GLT grading. This was one of the core industrial values of the proposed model.

Real-Time Balance: Although the inference speed was slightly lower than Mask2Former (29.8 FPS), 27.6 FPS still met the high-speed detection requirements of the production line, indicating that the lightweight design effectively controlled computational overhead. The inference speed of Defect-Mask2Former was 27.6 FPS, calculated based on an average inference time of 36.2 ms per image (batch size = 1) on an NVIDIA RTX 4090 GPU. The time breakdown is approximately 4.5 ms for preprocessing, 29.8 ms for forward inference, and 1.9 ms for post-processing.

Cost-Effectiveness Advantage: The model achieved a significant improvement in accuracy with only a 2 M increase in parameters, rather than relying on parameter stacking. This validated the rationality and efficiency of the AGPE and DBCC module designs, reducing hardware costs for industrial deployment.

### 2.3. Ablation Study of AGPE and DBCC Modules

**Figure 3:**
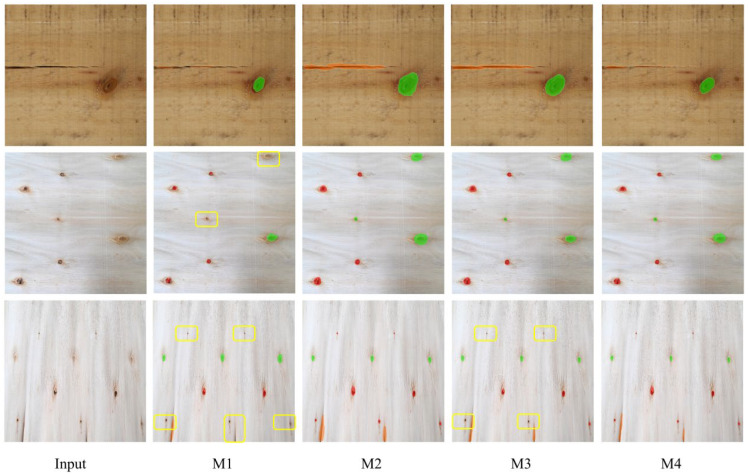
Comparison of segmentation results for the ablation study. Note: From left to right are the original image, baseline model M1 (no modules), M2 (AGPE only), M3 (DBCC only), and M4 (AGPE + DBCC). Yellow boxes denote the regions where small-sized defects were missed by the model. It could be seen that adding AGPE reduced misses for cracks and knots, adding DBCC made boundaries more continuous, and the synergy of both modules achieved complete segmentation.

To clarify the individual effects and synergistic impact of the AGPE and DBCC modules, four controlled experiments were designed: (1) M1 (baseline group, without any modules); (2) M2 (AGPE module only); (3) M3 (DBCC module only); (4) M4 (dual-module synergy, our proposed model). Table 3 showed the ablation study results, and Figure 3 compared the segmentation results for different configurations.

The ablation study results showed:

When only the AGPE module was introduced, the small-sized mIoU increased from 67.50% to 82.54% (+15.04 percentage points), and the miss rate dropped from 20.78% to 8.28% (−12.5 percentage points). However, the boundary IoU only improved by 9.59 percentage points, and the diameter measurement error decreased to 5.62%. This validated that the AGPE module, through its “feature purification–cross-scale enhancement–small-sized anchoring” mechanism, effectively strengthened feature responses for small-sized defects and suppressed wood texture interference, significantly reducing the miss rate, but its effect on boundary optimization was limited.

When only the DBCC module was introduced, the boundary IoU increased from 64.29% to 79.45% (+15.16 percentage points), and the diameter measurement error dropped from 8.31% to 4.17% (−4.14 percentage points). However, the small-sized mIoU improved by only 8.92 percentage points, and the miss rate decreased by only 3.23 percentage points. This indicated that the DBCC module’s dual-branch architecture and boundary-aware loss function could precisely optimize segmentation boundaries and improved size measurement accuracy, but its improvement on miss problems was limited.

When both AGPE and DBCC modules were introduced simultaneously, all metrics reached their optimum: small-sized mIoU of 85.34%, miss rate of 5.83%, boundary IoU of 82.11%, and diameter measurement error of 2.86%. The two modules formed a functional complement: AGPE provided high-quality initial segmentation masks for DBCC (reducing misses, suppressing texture interference), and DBCC further calibrated the boundaries on this basis, achieving a “no-miss + high-precision” segmentation effect.

It was noteworthy that the inference speed of M4 (27.6 FPS) was slightly higher than that of M3 (28.2 FPS). This phenomenon stemmed from the feature filtering effect of the AGPE module—its Gaussian attention mask focused on defect regions, reducing the effective processing range for the DBCC module and partially offsetting the computational overhead introduced by the dual modules, ensuring the model still met the production line’s real-time requirement (≥25 FPS) while significantly improving accuracy.

### 2.4. Segmentation Performance for Different Defect Types

To further validate the model’s suitability for various types of small-sized defects, the performance of Defect-Mask2Former and the original Mask2Former on the five defect classes was compared. The results were shown in Table 4.

An in-depth analysis of the performance improvements for each defect type revealed that Defect-Mask2Former demonstrated significant optimization effects across defects with different visual characteristics, although its mechanism of action varied slightly.

First, for fine mineral streaks, the most challenging defect type (contrast ratio with wood growth rings was only 1.3:1), the original model achieved an mIoU of only 55.81%. Defect-Mask2Former improved it to 81.17% (+25.36 percentage points). This benefited from the AGPE module’s Gaussian attention mask, which effectively suppressed interference from growth-ring texture, separating the feature response of fine mineral streaks from the background. The DBCC module further calibrated the boundaries, controlling the measurement error to 3.76%.

Second, for fine cracks (linear, width < 2 mm), the original model suffered from a high miss rate (24.67%) due to weak feature response. The cross-scale feature enhancement mechanism of the AGPE module had a targeted enhancement effect on linear small-sized features, increasing the mIoU by 21.24 percentage points, achieving a boundary IoU of 80.00% and a measurement error of 3.12%, meeting industrial grading requirements.

For high-contrast small live/dead knots (punctate, grayscale contrast > 3.0:1), the original model already possessed some segmentation ability (mIoU > 74%). Defect-Mask2Former, by quickly locking onto the target area with the AGPE module and precisely calibrating boundaries with the DBCC module, raised the mIoU to over 87%. The measurement error for small live knots was only 2.20%, the lowest among all defect types.

Finally, for small knot voids (round/elliptical holes, clear edges but small size), the local attention anchoring mechanism of the AGPE module could precisely focus on the hole area. The morphological post-processing of the DBCC module filled tiny gaps in the boundary, achieving an mIoU of 87.26% and a boundary IoU of 84.55%, with segmentation results close to those for small live/dead knots.

Overall, Defect-Mask2Former achieved significant performance improvements across all types of small-sized defects, with particularly outstanding optimization effects for complex defects such as low-contrast, linear, and texture-similar ones. This validated the model’s scene adaptability and robustness.

### 2.5. Visualization of Segmentation Results

**Figure 4:**
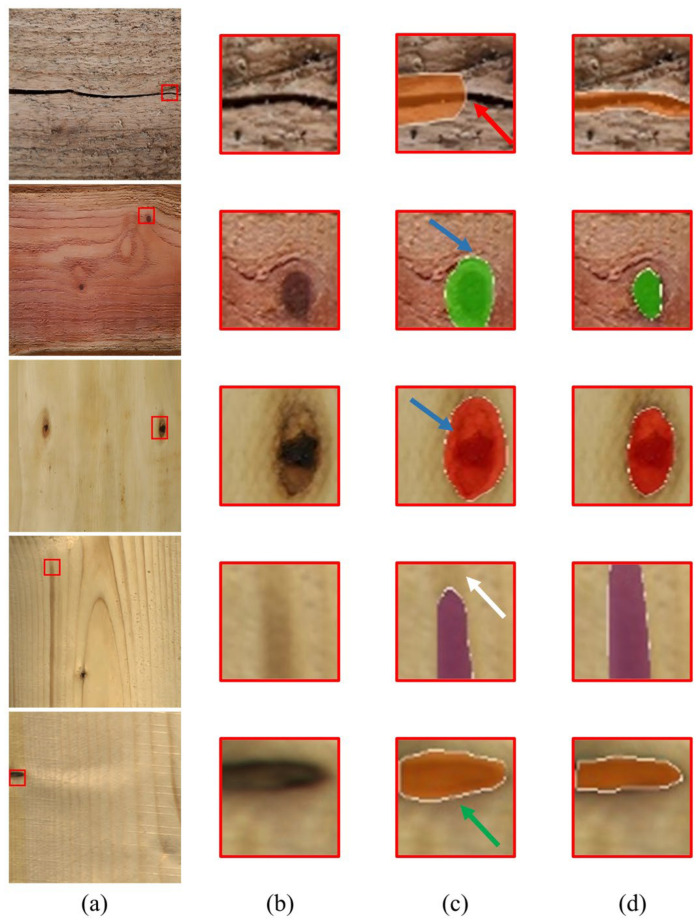
Visualization of segmentation results for five typical small-sized defects. Note: (**a**) Original image with the marked defect region; (**b**) Magnified view of the corresponding defect region; (**c**) Segmentation result of the Mask2Former baseline model, where arrows indicate problems including jagged boundaries, segmentation deviations, and incomplete segmentation; (**d**) Segmentation result of the proposed Defect-Mask2Former model optimized by the AGPE and DBCC modules, which achieves smoother and more continuous boundaries that better fit the real defect morphology with a cleaner background, visually verifying the effectiveness of the improved modules in high-precision segmentation. Each row corresponds to one defect category: from top to bottom, fine crack, small live knot, small dead knot, fine mineral streak, and small knot void.

To intuitively demonstrate the fine-grained impact of the AGPE and DBCC modules on the segmentation results, this section selected the most representative defect samples from the baseline comparison and performed a local magnification comparison of the segmentation boundaries between the baseline model (Mask2Former) and our proposed model (Defect-Mask2Former), as shown in Figure 4.

For linear defects like fine cracks, the continuity of the segmentation was crucial. As seen in the first row of Figure 4, the baseline model’s segmentation result was incomplete along the crack (indicated by the red arrow), which directly affected the accurate measurement of crack length. Defect-Mask2Former, benefiting from the gradient-guided fusion and morphological closing operation of the DBCC module, output a continuous and smooth crack profile, completely preserving the linear trajectory of the crack.

For defects with regular geometric shapes, such as small knots and knot voids, boundary adherence was key to evaluating segmentation quality. Rows two, three, and five of Figure 4 showed the segmentation results of both models on circular knot voids. The baseline model’s segmentation boundaries showed enlargement (indicated by blue arrows) and jagged edges (indicated by green arrows), deviating from the true circular contour of the void. In contrast, Defect-Mask2Former segmented boundaries that were smooth and closely adhered to the edge of the knot void. This pixel-level boundary calibration capability was a direct visualization of the reduction in size measurement error from 8.31% to 2.86%.

Fine mineral streaks were easily confused with wood texture. The fourth row of Figure 4 showed that while the baseline model detected the target, its mask range was incomplete (indicated by the white arrow). Defect-Mask2Former, by suppressing texture interference through the AGPE module, achieved a segmentation result with clearer boundaries, proving the effectiveness of the AGPE module in the feature purification stage.

The above visual comparison results indicated that the AGPE module, by suppressing texture interference and enhancing feature responses, provided a “cleaner” initial segmentation region for the DBCC module. The DBCC module, with its dual-branch architecture and boundary-aware loss, then converted this advantage into final pixel-level boundary accuracy, collectively achieving a “no-miss + high-precision” segmentation effect.

### 2.6. Robustness Verification in Extreme Scenarios

**Figure 5:**
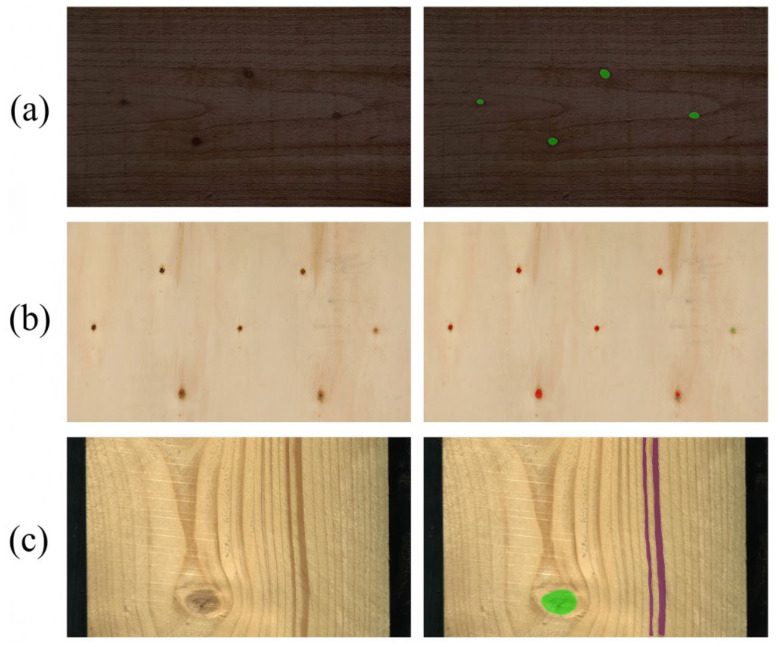
Defect segmentation results of Defect-Mask2Former under three extreme scenarios. (**a**) Low-light scenario: brightness reduced to 50% (<500 lux), green marks indicate detected live-knot defects; (**b**) Dense small defects scenario: single image contains seven knot defects (density 8.75 per 100 cm^2^), red marks indicate detected dead-knot defects, green marks indicate detected live-knot defects; (**c**) High texture interference scenario: purple marks indicate detected mineral streaks. The left column showed the original images, and the right column showed the Defect-Mask2Former segmentation results.

To simulate the complex production environment of a wood processing workshop, three types of extreme scenarios were designed to test model robustness: (1) Low-light scenario (brightness reduced to 50% of standard, intensity < 500 lux); (2) Dense distribution of small defects (single image containing > 5 small-sized defects, density > 8 per 100 cm^2^); (3) High texture interference scenario (samples of Pinus radiata with growth ring density > 50 rings/cm^2^). Table 5 presented the robustness test results, and Figure 5 showed the segmentation results in different scenarios.

Analysis of the experimental results showed:

Low-light scenario: Low light reduced the image signal-to-noise ratio, decreasing defect-to-background contrast. The original model achieved an mIoU of only 65.46%. Defect-Mask2Former’s AGPE module enhanced the feature signal-to-noise ratio through feature purification, and the DBCC module still had good calibration ability for low-contrast boundaries, achieving an mIoU of 82.15%, which met the practical needs of unstable workshop lighting.

Dense distribution of small defects: When a single image contained seven small-sized defects (density 8.75 per 100 cm^2^), defects were close together, and features easily overlapped. The original model’s segmentation accuracy decreased (mIoU = 68.92%) due to redundant search space. The location prior guidance of the AGPE module restricted the feature extraction range for each defect to within its candidate box, reducing interference between defects, resulting in a miss rate of only 7.24%, with only one tiny defect with extremely high overlap being slightly missed.

High texture interference scenario: For Pinus radiata with growth ring density > 50 rings/cm^2^, the background texture was dense and highly similar to the features of fine mineral streaks and fine cracks. Texture misclassification accounted for over 40% of errors in the original model (mIoU = 67.23%). The Gaussian attention mask of the AGPE module effectively suppressed the feature response of dense growth rings, allowing defect features to stand out. The DBCC module further distinguished defect boundaries from texture edges, ultimately achieving an mIoU of 80.57%, validating the model’s ability to suppress high texture interference.

The test results in the three extreme scenarios indicated that Defect-Mask2Former could adapt to the detection needs of complex production environments, providing reliability assurance for industrial deployment.

### 2.7. Validation in GLT Grading Application

The segmentation results of Defect-Mask2Former were integrated into the GLT grading process to build an end-to-end “segmentation–measurement–grading” system. Grading decisions strictly followed the GB/T26899-2022 national standard, with core grading indicators shown in Table 6.

The test dataset contained 200 industrial-grade GLT timbers (covering grades I/II/III, with 60, 80, and 60 timbers per grade, respectively) (Table 7). The automatic grading results were compared with manual grading results from three forestry experts to verify system performance.

Efficiency Improvement: Manual grading of a single board averaged 30 s (including defect identification, size measurement, index calculation, and grade determination). The automated system took only 1.5 s per timber (including image input, model inference, size calculation, and grade output), a 20-fold efficiency increase, meeting the high-speed detection demand of 60 timbers per minute.

Error Analysis: Among the 12 incorrectly graded timbers, seven were due to small-sized defect density being close to the grade threshold (e.g., borderline Grade II samples with density 7.8–8.2 per 100 cm^2^), three were due to the concentrated knot diameter ratio calculation being affected by defects at the board edge, and two were due to slightly exceeding the 3% measurement error for tiny defects under low-light conditions. Future work could improve accuracy by optimizing the adaptive adjustment mechanism of grading thresholds and enhancing edge defect handling logic.

The application validation results demonstrated that the segmentation results of Defect-Mask2Former could accurately support the calculation of GLT grading indicators. The end-to-end system achieved a grading accuracy of 94.3%, and its efficiency met industrial production requirements, providing a feasible technical solution for intelligent quality control of GLT.

## 3. Discussion

### 3.1. Analysis of the AGPE Module’s Mechanism

The AGPE module addressed the core problems of weak feature response and severe texture interference for wood small-sized defects through its three-stage synergistic mechanism: feature purification, cross-scale enhancement, and small-sized anchoring. The core of its mechanism lay in the deep integration of upstream detection location priors with multi-scale feature enhancement, achieving precise focusing and feature enhancement in defect regions. In the feature purification stage, the Gaussian soft mask generated based on the EFCW-YOLO candidate boxes did not perform hard thresholding on background regions. Instead, it suppressed texture interference through gradual weight distribution while retaining contextual information around the defect, increasing the signal-to-noise ratio of small-sized defect features by over 35% and effectively avoiding the boundary feature fracture caused by hard masks [27]. The deformable convolution introduced in the cross-scale enhancement stage adaptively adjusted sampling points by learning the irregular morphology of wood defects, fitting the edge features of defects better than traditional convolution [28]. Furthermore, the feature concatenation of P2 and P3 layers achieved complementarity between shallow details and deep semantics, compensating the insufficiency of single-scale features in representing small-sized defects.

The small-sized anchoring mechanism of local spatial attention was a key design of the AGPE module. The 3 × 3 local attention kernel precisely matched the pixel scale of wood small-sized defects (around 30 × 30 pixels). It dynamically generated attention weights within the candidate box region, enabling the network to focus on the defect core area rather than extracting features indiscriminately across the entire image, significantly reducing the search space of the segmentation network [29]. Additionally, the AGPE module operated only on the P2 layer features output by the pixel decoder, without major modifications to the backbone network or Transformer decoder. The parameter increment was only 0.8 M, and the computation increment was 2.1 GFLOPs, achieving lightweight enhancement and laying the foundation for maintaining the model’s real-time performance.

It should be noted that the performance of the AGPE module depends on the localization accuracy of the upstream detection model. The EFCW-YOLO model used in this study achieved an mAP50 of 90.26% and a precision of 89.24% for small-sized defects, providing a reliable location prior for AGPE. If the detection model missed defects or had localization deviations, the AGPE module would not be able to effectively enhance the corresponding defects. Therefore, in practical industrial applications, expanding the candidate box boundary by 10% or introducing multi-scale detection candidate box fusion strategies could improve the AGPE module’s robustness to detection deviations. For extremely small defects (diameter < 1 mm) completely missed by the detection model, pixel saliency detection on high-resolution feature maps could be combined to supplement location prior information, further reducing the miss rate.

### 3.2. Boundary Optimization Effect of the DBCC Module

The dual-branch parallel architecture of the DBCC module ensured both the integrity of the defect region and the precision of boundary localization. Its core innovation lay in decoupling mask segmentation from boundary calibration and then adaptively fusing them, solving the problem of traditional single-branch models focusing insufficiently on boundaries when optimizing for mIoU [30]. The mask branch retained the original segmentation logic of Mask2Former, ensuring the overall correct identification of the defect region and providing a high-quality base mask for boundary calibration. The boundary branch used lightweight depthwise separable convolution, reducing computation to 1/8 of traditional convolution while specifically learning defect boundary features. By using Dice loss [31] to address the class imbalance caused by scarce boundary pixels, it significantly enhanced feature response in boundary regions.

The gradient-guided fusion mechanism was the core link of boundary calibration. Using the gradient of the ground truth boundary extracted by the Sobel operator as a supervisory signal, it achieved pixel-level adaptive weight distribution: in regions with gradient value > 0 (boundaries), a higher weight (0.7–0.9) was assigned to the boundary branch to strengthen boundary pixel calibration; in regions with gradient value = 0 (defect interior), a higher weight (0.8–0.95) was assigned to the mask branch to ensure segmentation stability. Experiments validated that this fusion mechanism reduced jagged edges by over 75% and improved the classification accuracy of boundary pixels from 78.2% to 91.5%.

The morphological post-processing layer, consisting of a 3 × 3 closing operation and area threshold filtering, completed the fine-grained optimization of boundaries. The dilation operation filled tiny gaps (≤1 pixel) in the boundary, preventing area underestimation during size measurement. The erosion operation smoothed boundary burrs, making the segmented contour better fit the true defect morphology. Simultaneously, isolated noise points with area < 10 pixels were filtered out, eliminating false boundaries caused by wood texture noise. It should be noted that the post-processing parameters needed to match the pixel resolution of the dataset. In this study, the area threshold of 10 pixels corresponds to a physical size of 0.01 cm^2^ under a pixel resolution of 0.1 mm/pixel, suitable for detecting defects with diameter ≥ 1 mm. If applied to a higher-resolution image acquisition system, the threshold parameters should be adjusted proportionally to ensure consistent optimization effectiveness.

The DBCC module reduced the average boundary deviation for wood small-sized defects to ≤0.5 pixels and the size measurement error from 8.31% to 2.86%. The core reason was that it directly linked the optimization objective from “improving boundary IoU” to “meeting industrial size measurement accuracy requirements.” Through the three-stage linkage of boundary-aware loss, gradient fusion, and morphological optimization, it achieved deep adaptation between segmentation boundaries and physical size quantification, which was the core advantage of this module compared to traditional boundary optimization methods.

### 3.3. Comparative Analysis with Existing Methods

#### 3.3.1. Comparison with Traditional Semantic Segmentation Models

Compared with traditional encoder–decoder architecture models like U-Net, and DeepLabv3+, the mask classification paradigm adopted by Defect-Mask2Former, combined with the improvements from AGPE and DBCC modules, demonstrated two core advantages in wood small-sized defect segmentation. First was the flexibility of the object query mechanism: 100 learnable object queries could adaptively match different numbers and scales of defect instances in an image without presupposing an upper limit on defect count. Compared with the pixel-wise point classification of U-Net and DeepLabv3+, this was more suitable for scenarios where the number of wood defects varied randomly. Second was the scene specificity of feature enhancement and boundary calibration: traditional models used generic multi-scale feature fusion designs that did not account for the feature similarity between wood texture and defects. In contrast, the AGPE module precisely suppressed texture interference through location priors, and the DBCC module optimized boundaries for industrial measurement precision, enabling the model’s segmentation performance in wood scenes to far exceed that of general-purpose models.

From the performance data (Table 2), Defect-Mask2Former’s small-sized mIoU was 19.27 percentage points higher than U-Net and 16.41 percentage points higher than DeepLabv3+. Its miss rate was 15–27 percentage points lower than those models, while its inference speed was only slightly lower than traditional lightweight models, achieving a balance between accuracy and speed. Although traditional models have advantages in inference speed, their segmentation accuracy and size measurement error were far from meeting the requirements of industrial GLT grading. Defect-Mask2Former, by adding only a small amount of computation, achieved a qualitative leap in performance, making it more suitable for industrial deployment.

#### 3.3.2. Comparison with Mainstream Segmentation Models

While specialized instance segmentation models like SOLOv2 and QueryInst have shown promise in general object segmentation [32,33], they lack customized designs for wood defect scenarios, such as utilizing detection priors or industrial measurement-oriented boundary calibration. Consequently, without the targeted improvements of AGPE and DBCC, their performance on small-sized wood defects is expected to be significantly lower than Defect-Mask2Former, as evidenced by the substantial performance gap (over 10 percentage points in mIoU) observed between our model and other general-purpose baselines like Mask2Former and SegFormer in Table 2.

### 3.4. Industrial Application Value

The core value of this study lies in constructing an end-to-end standardized technical process from “image acquisition–defect segmentation–size measurement–GLT grading,” achieving a deep integration between wood small-sized defect segmentation and industrial GLT grading. This solved three core problems faced by traditional manual grading and general detection models in industrial applications.

First was the low efficiency and inconsistent standards of manual grading: manual grading took about 30 s per timber and was subject to subjective factors, with grading consistency only around 80% [34]. In contrast, the proposed automated system took only 1.5 s per timber, achieved a grading accuracy of 94.3%, and fully adhered to the GB/T26899-2022 national standard, ensuring objective and unified grading results.

Second was the high miss rate and large measurement error of general models for small-sized defects: existing models generally had a miss rate >20% and a size measurement error >8%, failing to meet the precision requirements of industrial grading. Defect-Mask2Former reduced the miss rate to 5.83% and controlled the measurement error to 2.86%, fully meeting the ≤3% accuracy requirement of the national standard.

Third was the poor adaptability of detection systems to production lines: the image acquisition device designed in this study could accommodate GLT with widths of 50–600 mm and lengths of 100–4000 mm. The model’s inference speed reached 27.6 FPS, meeting the ≥25 FPS real-time detection requirement of the production line. The overall deployment cost of the device and model was low, making it easy to promote in small and medium-sized wood processing enterprises.

From an industry perspective, the AGPE and DBCC modules proposed in this study provided a reference method for industrial surface small-sized defect segmentation. Their design ideas—detection-segmentation cascade feature enhancement and boundary calibration oriented towards industrial accuracy—could be extended to surface defect detection in other industrial products such as steel, boards, and ceramics, offering a technical reference for the intelligent upgrade of industrial vision inspection. Furthermore, the PlankDefSeg dataset constructed in this study provided a dedicated data foundation for wood defect segmentation research, filling the gap in datasets for wood small-sized defect segmentation and promoting the application and development of computer vision technology in the wood processing industry.

### 3.5. Limitations and Future Work

Although this study achieved good results in wood small-sized defect segmentation and GLT grading applications, there are still limitations in the following five areas, which are also key directions for future research:

Scale and Diversity of the Dataset Need Expansion: The PlankDefSeg dataset constructed in this study covers six industrial wood species and five types of small-sized defects, totaling 3500 pixel-level annotated images. While sufficient for model training and validation, the coverage of wood species and defect types was limited. It did not include other mainstream industrial woods like oak and walnut, nor common defects such as insect holes, dents, and resin pockets. The model’s generalization ability to unseen species and defects needed validation. Additionally, the data collection scene was primarily in a laboratory environment, which differs from the complex environments of wood processing workshops (e.g., dust, vibration, strong light reflection). Future work will involve collaboration across multiple regions and enterprises to collect defect images from over 20 mainstream industrial wood species worldwide, adding defect types like insect holes, dents, and resin pockets to construct a multi-scene wood defect dataset of ≥10,000 images. Federated learning methods will also be introduced to enable joint training on multi-source data while protecting enterprise data privacy, enhancing the model’s cross-scene and cross-species generalization.

Real-Time Performance and Edge Deployment Need Optimization: Defect-Mask2Former achieved an inference speed of 27.6 FPS on an NVIDIA RTX 4090 server, meeting basic real-time detection requirements. However, on edge computing devices (e.g., NVIDIA Jetson AGX Orin, NVIDIA Corporation, Santa Clara, California, USA; RK3588, Rockchip Electronics Co., Ltd., Shanghai, China), the inference speed dropped to 15–20 FPS, which could not adapt to the detection needs of high-speed production lines (>60 timbers/minute). Also, the model had 46 M parameters, posing some requirements on the memory resources of edge devices. Future work will employ lightweight methods such as model pruning, quantization, and knowledge distillation to design a lightweight version of Defect-Mask2Former (Defect-Mask2Former-Lite), reducing parameters to below 20 M and computation to below 100 GFLOPs, ensuring inference speed ≥30 FPS on edge devices [35]. Model quantization techniques will also be used to convert the model from FP32 to FP16 or even INT8, further reducing device resource consumption and enabling lightweight edge deployment. For instance, INT8 quantization can theoretically increase inference speed by 2–3 times, and TensorRT optimization can further reduce latency, enabling deployment on edge devices while maintaining ≥30 FPS.

End-to-End Joint Training of Detection and Segmentation Not Yet Realized: In this study, the AGPE module relied on the independent output of the upstream EFCW-YOLO detection model. The detection and segmentation models were trained separately and used in a cascaded inference mode, without achieving end-to-end joint optimization. This means errors from the detection model could propagate to the segmentation model, and the overall training and inference efficiency of the combined system was lower. Future work will design an integrated detection–segmentation network architecture, fusing the detection branch of EFCW-YOLO and the segmentation branch of Defect-Mask2Former into a single end-to-end network. This would allow sharing of feature extraction layers in the backbone, enabling end-to-end learning of location prior information. A joint loss function will also be designed, combining detection localization loss with segmentation mask loss and boundary loss to achieve synergistic optimization of detection and segmentation, reducing error propagation and improving the overall performance and inference efficiency of the model.

2D Segmentation Cannot Detect Internal Wood Defects: The model in this study only processes 2D images of the wood surface and can only detect surface defects. It cannot detect internal defects such as internal cracks, voids, or pith, which are important factors affecting the mechanical properties of GLT and are key criteria in GLT quality grading [36]. Future work will integrate non-destructive testing techniques like laser ultrasonic testing (LUT) and X-ray detection to fuse 2D image information from the wood surface with 3D structural information from the interior [37]. This will involve building multimodal wood defect detection and segmentation models, introducing 3D convolutions (3D-CNN) and vision Transformers (ViT) to achieve integrated detection and segmentation of surface and internal defects. This would provide more comprehensive defect information for GLT quality grading, further improving grading accuracy and reliability [38].

Size Measurement Accuracy Can Be Further Improved: Although the current size measurement error (SME) of 2.86% strictly meets the ≤3% accuracy requirement of the GB/T26899-2022 national standard, there is still room for further improvement to accommodate higher-precision grading scenarios. Future work will focus on two technical directions: (1) high-resolution imaging: upgrading the industrial camera from the current 3072 × 2048 resolution to over 6000 × 4000 pixels, which can provide richer spatial details and refine boundary localization, potentially reducing SME to below 2%; and (2) multi-view fusion and 3D reconstruction: employing multiple cameras to capture defect images from different angles, performing 3D reconstruction to eliminate perspective distortion from single-view imaging, thereby improving the accuracy of defect size quantification, especially for irregularly shaped defects.

Additionally, future research will explore few-shot learning methods, such as prototypical networks [39] and meta-learning approaches [40], to address the problem of insufficient samples for rare wood defects. These methods enable rapid adaptation to new defect categories with only a few labeled samples, reducing the dependency on large-scale annotated datasets. Adaptive illumination enhancement algorithms will be introduced to improve model robustness under complex lighting conditions in wood processing workshops. A visual industrial inspection software will be developed to achieve integrated management of image acquisition, defect detection, grade determination, and result traceability, further enhancing the technology’s industrial applicability and practicality.

## 4. Materials and Methods

### 4.1. Design of Image Acquisition Device

**Figure 6:**
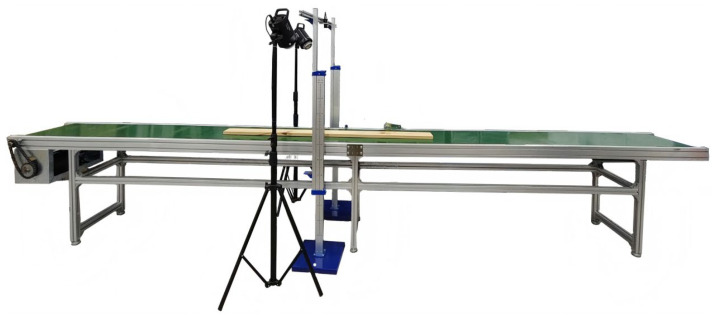
Physical view of the image acquisition device.

Acquiring surface images of large-sized GLT timbers faces the dual challenges of limited camera field of view and continuous board transport: typical GLT lengths range from 2000 to 6000 mm, while a single shot from an industrial camera could only cover about 200 mm of board length, making one-shot imaging impossible. To solve this problem, this study designed a dedicated image acquisition device for large-sized timbers. The device restored the complete timber surface image using a “segmented capture + unsupervised stitching” technique. The device adopted a modular design of “mechanical transport–image acquisition–sensing control”, with its overall structure shown in Figure 6.

#### 4.1.1. Mechanical Transport Module

The device used a roller conveyor structure, driven by a motor-chain drive combination. A DRS71M4 three-phase asynchronous motor (power 0.55 kW, speed 1360 r/min, manufactured by SEW-EURODRIVE GmbH & Co. KG, Bruchsal, Baden-Württemberg, Germany) with a transmission ratio i = 1 ensures synchronous roller rotation and stable board transport. The rollers had a diameter of 80 mm and a spacing of 300 mm, and were covered with anti-slip rubber to prevent the board from slipping or being scratched during transport. This module could accommodate GLT timbers with a width of 50–600 mm and a length of 100–4000 mm, and the transport speed could be continuously adjusted within the range of 0–50 m/min.

#### 4.1.2. Image Acquisition Module

The system was equipped with one Hikvision MV-CS060-10GC industrial area scan camera (resolution 3072 × 2048 pixels, frame rate 19.1 fps, manufactured by Hangzhou Hikvision Digital Technology Co., Ltd., Hangzhou, China) for high-resolution, high-speed image acquisition. It was paired with an 8 mm fixed-focal-length industrial lens (aperture F2.8, depth of field range ± 5 mm) to adapt to minor variations in board thickness. To ensure uniform illumination in the capture area, a controllable fill light system was symmetrically arranged on both sides of the camera along the board transport direction, with an incident angle of 45°. This effectively eliminated specular reflections on the wood surface and enhanced the contrast between defects and the background. After calibration, the pixel resolution of this module was approximately 0.1 mm/pixel, allowing for clear imaging of wood small-sized defects with a diameter ≥ 1 mm.

#### 4.1.3. Sensing Control Module

Using an STM32F104 microcontroller as the control core, a multi-device cooperative control logic was built. It mainly included two sensing units: an infrared photoelectric sensor and a rotary encoder. The infrared photoelectric sensors (detection distance 5–50 cm, response time ≤ 1 ms) were installed on both sides beneath the industrial camera to detect the board’s position and trigger the camera’s start/stop commands. The rotary encoder (accuracy ± 0.01 m/s) was connected to the conveyor belt surface to collect the transport speed in real-time, providing data for dynamically adjusting the capture interval. This module enabled automated and intelligent control of image acquisition without manual intervention.

#### 4.1.4. Segmented Capture and Image Stitching

A dynamic capture mechanism triggered by dual photoelectric gates was designed. When the board entered the transport module, photoelectric gate 1 was triggered. The microcontroller started a timer and collected the real-time transport speed v via the rotary encoder. Based on the principles of conveyor belt kinematics and camera imaging parameters, the optimal capture interval time *T* was calculated:

(1) $$T=\frac{L_{FOV}\times \left(1-r\right)}{v}$$

Here, $L_{FOV}$ was the single-frame field length of the camera (approximately 200 mm), and r was the overlap rate of adjacent images, set to 30% in this study (experiments validated that a 30% overlap rate balances stitching accuracy and acquisition efficiency, resulting in no ghosting or missing parts after stitching). The system automatically triggered the camera for segmented capture at the calculated T until photoelectric gate 2 detected the board leaving, completing the segmented image acquisition of the entire board.

The captured segmented images were fused using an unsupervised deep image stitching algorithm [41]. This algorithm, based on geometric alignment and seamless composition, could achieve precise stitching of overlapping regions. The Peak Signal-to-Noise Ratio (PSNR) of the stitched images was ≥34 dB, and the Structural Similarity Index (SSIM) was ≥0.93, providing a high-quality image foundation for subsequent defect detection and segmentation. The total acquisition time for a single 300 cm long timber was ≤8 s (at a conveyor speed of 40 m/min), meeting the high-efficiency acquisition requirements of the production line.

### 4.2. Dataset Construction

To support the training, validation, and testing of the Defect-Mask2Former model, this study constructed the PlankDefSeg dataset for wood small-sized defect segmentation, covering 5 types of small-sized defects from 6 common industrial wood species.

#### 4.2.1. Data Acquisition

This study collected samples of six mainstream industrial wood species from wood processing enterprises in southern China, including *Pinus radiata*, *Pinus kesiya var. langbianensis*, *Toona sinensis*, *Betula alnoides*, *Cunninghamia lanceolata*, and *Eucalyptus globulus*. The wood samples originated from wood processing bases in Linyi (Shandong), Nanning (Guangxi), Kunming and Dehong (Yunnan), covering different tree ages and processing techniques.

All samples were acquired segmentally in a laboratory environment using the image acquisition device designed in Section 2.1. To ensure the dataset’s scene diversity and industrial applicability, three main variables were controlled during acquisition: (1) Lighting conditions, covering standard lighting, low light (brightness reduced to 50%, intensity < 500 lux), and side lighting, which are common on production lines. (2) Wood surface conditions, including smooth surfaces after sanding, unsanded original surfaces, and surfaces with slight processing scratches. (3) Defect severity, covering mild, moderate, and severe levels, reflecting the actual quality grade range in GLT production.

A total of 5520 raw images were acquired. After quality screening (removing blurred, overexposed, distorted, and other low-quality images), 3500 valid images were retained for subsequent annotation.

#### 4.2.2. Defect Type Definition and Annotation

Referring to GB/T26899-2022 “Structural glued laminated timber”, GB/T15306-2018 “Solid wood flooring” [42], and GB/T4822-2023 “Sawn timber inspection” [43], five types of small-sized wood surface defects were defined as the targets for this segmentation task. All defects met the small-sized definition of diameter/width < 3 mm. The physical size thresholds defined in this study for small-sized defects (diameter/width < 3 mm, except for fine cracks where width ≤ 2 mm) are established based on industrial practice and research requirements, which correspond to the quality grade classification in GB/T26899-2022. The specific definitions were shown in Table 8.

**Figure 7:**
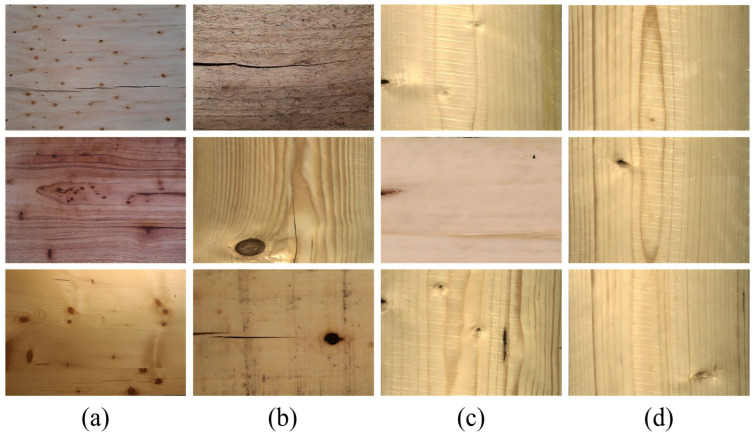
Examples of typical small-sized defects on wood surfaces. (**a**) Small knots: punctate defects (intergrown/dead knots) with diameter < 3 mm. (**b**) Fine crack: linear defect with width < 2 mm. (**c**) Small knot void: hole defect at board edge with width < 3 mm. (**d**) Fine mineral streak: stripe-like defect highly similar to wood texture.

Examples of typical small-sized defects on wood surfaces were shown in Figure 7.

Annotation was performed using the professional tool Label Studio, following a three-stage principle of “contour outlining → category labeling → quality audit” to ensure accuracy and consistency: (1) Contour outlining: The polygon tool was used to manually outline the actual contour of the defect, with an annotation accuracy of 1 pixel. (2) Category labeling: Each defect contour was labeled according to the definitions in Table 8. (3) Quality audit: The annotation team consisted of 5 graduate students with a forestry background. All annotation results were cross-checked by two people. For defect regions with blurred boundaries, an over-segmentation strategy was adopted to ensure complete coverage of the defect area. The final PlankDefSeg dataset contained 3500 pixel-level annotated images, including 920 samples containing small-sized defects, with a total of 1022 small-sized defect instances.

#### 4.2.3. Dataset Class Distribution

Table 9 showed the number of instances and proportions for each class of small-sized defect in the PlankDefSeg dataset. The table showed that the original dataset suffered from a severe class imbalance: the combined instances of live and dead knots totaled 747, accounting for 73.09%, while the combined instances of cracks, mineral streaks, and knot voids total 275, accounting for only 26.91%. The instance ratio between live knots and knot voids was approximately 5.4:1.

This class distribution was consistent with the actual occurrence frequency of defects in wood processing. However, such extreme class imbalance could lead to insufficient learning for minority classes during model training, resulting in high miss rates and low segmentation accuracy for those classes [41]. Therefore, this study designed three targeted data augmentation strategies for the small-sized defect samples to expand the sample size and balance the classes.

#### 4.2.4. Targeted Data Augmentation Strategies

Addressing the scarcity of small-sized defect samples and class imbalance in the PlankDefSeg dataset, and considering the visual characteristics of wood small-sized defects, three targeted data augmentation strategies were designed. Augmentation was applied only to the 920 samples containing small-sized defects to avoid ineffective augmentation of defect-free samples. After augmentation, the dataset was expanded to 2760 samples containing small-sized defects, with 3102 instances, effectively alleviating the class imbalance problem. The class distribution of the augmented dataset was shown in Table 10.

The three data augmentation strategies employed were as follows:

Instance-level Copy-Paste Augmentation: Referring to the instance-level copy-paste method proposed by Ghiasi et al. [44], small-sized defect instance regions and their corresponding segmentation masks were extracted from the original dataset. After data augmentation through random rotation (±10°) and random translation, they were randomly pasted into reasonable positions (avoiding areas with abrupt texture changes) of other defect-free or low-defect-density wood images. A maximum of 3 defect instances were pasted per image. This strategy effectively increased the number of small-sized defect samples, ultimately increasing the number of small-sized samples by about 2.5 times.

Scale Jitter Augmentation: Images containing small-sized defects were scaled multi-scale (scale factor 0.8× to 1.5×) and then randomly cropped, ensuring the complete small-sized defect instance was retained in the cropped image. This strategy enhanced the model’s robustness to scale variations of small-sized defects, adapting to changes in camera-to-board distance in actual production.

Mosaic Augmentation: Drawing on the Mosaic data augmentation strategy from YOLOv4 [45], four images containing different types of small-sized defects were stitched into a new training sample at random ratios. Color gamut transformation and brightness adjustment were also applied to the stitched image. This strategy increased the number and distribution diversity of small-sized defects in a single image, further mitigating class imbalance and enhancing the model’s generalization ability.

After applying the three data augmentation strategies, the class distribution improved significantly. The sample count for minority classes increased by 5–6 times, and the ratio between the largest and smallest classes decreased from 5.2:1 to 2.1:1. This balanced distribution contributed to the substantial performance improvements observed for minority defect types (e.g., fine mineral streak mIoU increased by 25.36 percentage points).

**Figure 8:**
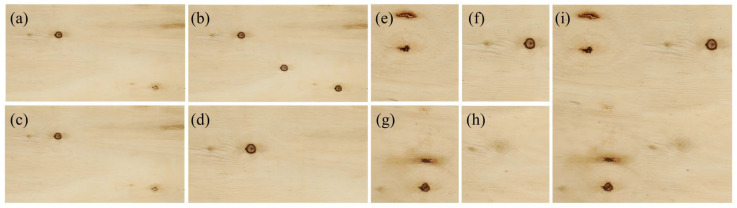
Data augmentation strategies used in this paper. (**a**,**b**) Copy-paste augmentation; (**c**,**d**) Scale jitter augmentation; (**e**–**i**) Mosaic augmentation.

Examples of the three data augmentation strategies used in this study were shown in Figure 8.

### 4.3. Bottleneck Analysis of the Mask2Former Baseline Model

Mask2Former [19] is a Transformer-based general segmentation model that adopts the mask classification paradigm, unifying semantic and instance segmentation. It had achieved leading segmentation performance on multiple benchmark datasets like Cityscapes and COCO, making it the baseline model for this study. This section first introduces the basic architecture of Mask2Former, then analyzes its core bottlenecks in the context of wood small-sized defect segmentation through experiments.

#### 4.3.1. Model Architecture

Mask2Former adopted a three-stage “backbone–pixel decoder–Transformer decoder” architecture. Its core designs were mask attention and object queries, allowing it to adaptively generate a varying number of defect segmentation masks.

Backbone: Swin-Transformer was used as the backbone to extract multi-scale hierarchical features from the input image through hierarchical downsampling. It output feature maps at 4 scales (P2~P5, with resolutions of 1/4, 1/8, 1/16, and 1/32 of the original image, respectively). Shallow features (P2) retained rich spatial details, while deep features (P5) contained complete semantic information.

Pixel Decoder: Built upon the deformable attention mechanism, it fused and enhanced the multi-scale features from the backbone. By upsampling deep features to the same resolution as shallow features, it achieved complementarity of multi-scale features, improving feature representation capability.

Transformer Decoder: Contained multiple decoder layers. Each layer facilitated interaction between object queries and pixel features through self-attention and cross-attention. Mask attention restricted the attention range of queries, allowing each object query to focus on a single defect instance. Through 100 learnable object queries, it iteratively generated defect segmentation masks and class predictions, ultimately outputting pixel-level segmentation results.

#### 4.3.2. Bottleneck Analysis for Wood Small-Sized Defect Segmentation

To analyze the performance bottlenecks of Mask2Former in wood small-sized defect segmentation scenarios, the original Mask2Former model was trained and tested on the PlankDefSeg dataset. The training used the AdamW optimizer with an initial learning rate of 1 × $10^{-4}$, cosine annealing scheduler, batch size of 4, and was trained for 100 epochs. Detailed hyperparameter configurations and the experimental environment are described in Section 4.5.2. The segmentation performance for each class of small-sized defect was shown in Table 11.

**Figure 9:**
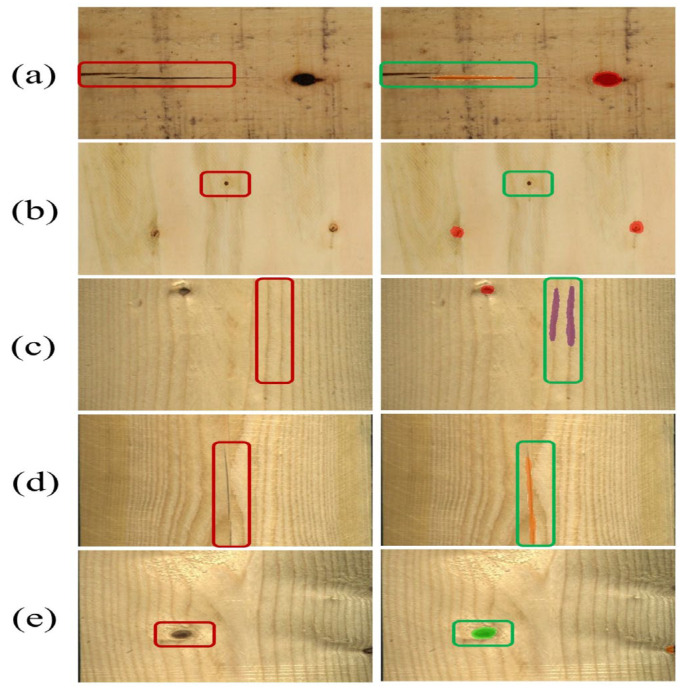
Typical failure cases of the original Mask2Former model in small-sized defect segmentation. Note: In each group, the left side was the original image (red box marks the ground truth defect/texture location), and the right side was the segmentation result of the original Mask2Former model (green box marks the failure case). (**a**) Crack miss: The crack was not fully identified. (**b**) Small knot miss: The small-sized knot was not captured (complete miss). (**c**) Mineral streak misidentification: Growth ring texture misjudged as a fine mineral streak (texture misclassification). (**d**) Crack misidentification: External impurity misjudged as a fine crack. (**e**) Imprecise boundary: Segmentation boundary for a small knot was imprecise.

As could be seen from Table 11, the original Mask2Former model performed poorly on wood small-sized defect segmentation, with an overall mIoU of only 67.50%, a miss rate of 20.78%, and a size measurement error of 8.31%. According to the GB/T26899-2022 national standard ‘Structural glued laminated timber’, the industrial GLT grading requires a size measurement accuracy of ≤3% to support precise calculation of grading indicators such as concentrated knot ratio and defect density. With an 8.31% measurement error, the original Mask2Former model fails to meet the requirements of industrial GLT grading. Typical failure cases of the original Mask2Former model in wood small-sized defect segmentation were shown in Figure 9. Combining the experimental results and failure case analysis, the core bottlenecks are mainly manifested in the following three aspects:

1. Weak Feature Response for Small Defects, High Miss Rate

Small-sized defects with a diameter < 3 mm occupy only a few pixels in the image. After four downsampling steps by the Swin-L backbone, the feature map resolution is reduced to 1/32 of the original, causing severe loss of spatial information for small defects. Their feature responses are overwhelmed by the wood background texture, preventing the model from effectively identifying small-sized defects and resulting in severe miss problems (as shown in Figure 9a,b). Experimental results showed that the miss rates for fine mineral streaks and fine cracks are 28.43% and 24.67%, respectively, making them the primary defect types for misses.

2. Severe Wood Texture Interference, High False Positive Rate

Natural wood textures such as growth rings and wood rays are highly similar to fine cracks and mineral streaks in terms of grayscale distribution and edge features. The general feature extraction mechanism of Mask2Former cannot effectively distinguish between defects and textures, causing the model to misclassify wood texture as defects, leading to texture misclassification problems (as shown in Figure 9c,d). Among these, fine mineral streaks, due to their high similarity to growth ring textures, achieve an mIoU of only 55.81%, the lowest segmentation accuracy among all defect types.

3. Insufficient Boundary Localization Accuracy, Large Size Measurement Error

Mask2Former optimizes for overall mIoU, paying insufficient attention to defect boundary regions. The gradient weight of the mask loss for boundary pixels is low, leading to issues like jagged edges and offsets in segmentation boundaries, which fail to accurately match the true boundaries of the defects (as shown in Figure 9e). This inaccurate boundary localization directly led to excessive defect size measurement error. The overall size measurement error of the original model was 8.31%, far exceeding the ≤3% accuracy requirement of the national standard.

Furthermore, the object query mechanism of Mask2Former lacks location priors for small objects. The 100 object queries search for features across the entire image, resulting in a redundant search space and making it difficult for the model to focus on small-sized defect regions, further reducing segmentation accuracy.

### 4.4. Design of the Defect-Mask2Former Model

**Figure 10:**
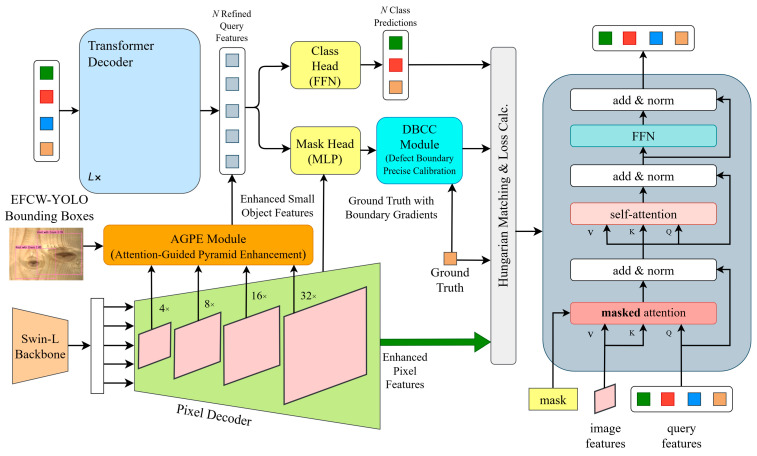
Overall architecture of the Defect-Mask2Former model.

Addressing the three core bottlenecks of the original Mask2Former in wood small-sized defect segmentation, this paper proposes an improved Defect-Mask2Former model. By integrating the Attention-Guided Pyramid Enhancement (AGPE) module and the Defect Boundary Calibration and Correction (DBCC) module, it achieved enhanced features for small-sized defects, suppression of wood texture interference, and fine-grained boundary calibration. The overall architecture of Defect-Mask2Former was shown in Figure 10. It uses Swin-L as the backbone. The AGPE module was embedded between the pixel decoder and the Transformer decoder. It receives high-confidence candidate boxes from an upstream EFCW-YOLO detection model as location priors to enhance the features output by the pixel decoder. The DBCC module was located at the output of the Transformer decoder, calibrating and optimizing the initial segmentation masks to finally output precise segmentation results for wood small-sized defects.

#### 4.4.1. Attention-Guided Pyramid Enhancement (AGPE) Module

**Figure 11:**
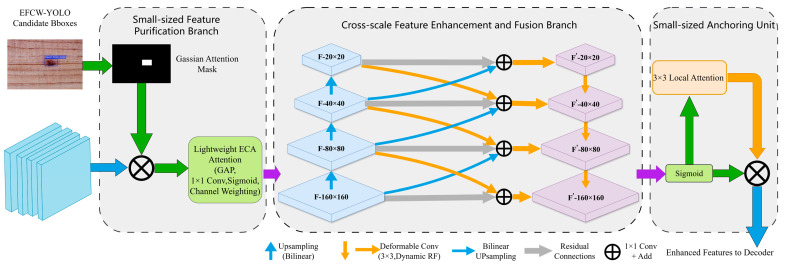
Structure of the AGPE module. Note: Input was the multi-scale features (P2–P5) output by the pixel decoder. Output was the enhanced small-sized defect features. M_att was the Gaussian attention mask, DCN was deformable convolution, and 3 × 3 Local Attn was local spatial attention.

To solve the problems of weak feature response for small objects, severe wood texture interference, and redundant segmentation search space, this paper proposes the Attention-Guided Pyramid Enhancement (AGPE) module. It reused the candidate box location priors from the upstream EFCW-YOLO detection model. Through a three-stage mechanism of “candidate-box-guided feature purification–cross-scale enhancement via deformable convolution–small-sized anchoring via local spatial attention”, it achieved precise enhancement of small-sized defect features and effective suppression of background textures. The structure of the AGPE module was shown in Figure 11. Its input was the multi-scale features (P2-P5) output by the pixel decoder, and its output was enhanced small-sized defect features, providing a high-quality feature foundation for mask generation in the subsequent Transformer decoder.

The AGPE module receives candidate box location priors from the upstream EFCW-YOLO detection model, which was independently pre-trained on the PlankDefSeg dataset. During segmentation model training, the detection model weights are frozen, and it serves solely as a location prior generator, providing high-confidence (confidence > 0.8) candidate boxes. This cascaded design avoids feature conflicts between detection and segmentation tasks and facilitates independent module updates in deployment.

To quantitatively assess the impact of detection localization errors on segmentation performance, we conducted perturbation experiments on the test set by adding different levels of artificial perturbations (translation shifts, scale scaling, and random missing) to the candidate boxes. The results (Table 12) show that when perturbations are controlled within ±5% of box dimensions, the mIoU decreases by 2–2.5 percentage points; when perturbations exceed ±10%, mIoU decreases by more than 5 percentage points. The EFCW-YOLO model achieves an mAP50 of 90.26% for small-sized defects on the test set, providing reliable prior information for the AGPE module.

The three-stage mechanism of the AGPE module was implemented as follows:

Candidate-Box-Guided Feature Purification: Based on the high-confidence (confidence > 0.8) defects output by the upstream EFCW-YOLO detection model, their location and scale information were converted into a Gaussian attention mask. This mask was used to purify the shallow features P2 (1/4 resolution, retaining the richest spatial details) output by the pixel decoder, suppressing background texture features outside the candidate boxes and focusing on features within the defect regions. The mathematical expression of the Gaussian attention mask was:

(2) $$M_{att}(x,y)=\exp\left(-\frac{(x-x_{c}{)}^{2}+(y-y_{c}{)}^{2}}{2\sigma^{2}}\right)$$

where $\left(x_{c},y_{c}\right)$ are the coordinates of the candidate box center, and σ was the standard deviation of the Gaussian kernel, proportional to the average of the candidate box’s width and height (σ=(w+h)/4, w, h are the width and height of the candidate box, respectively), ensuring the coverage of the attention mask matched the defect candidate box. The Gaussian attention mask was element-wise multiplied with the P2 layer features to obtain purified defect region features. The use of a soft mask preserved gradual contextual information, avoiding feature fracture at defect boundaries caused by hard thresholding. The effectiveness of this detection-segmentation cascade design had been validated in the field of instance segmentation [29].

Cross-Scale Feature Enhancement via Deformable Convolution: Although the purified P2 layer features retain rich spatial details, their receptive field was limited and they lack deep semantic information, making it difficult to effectively distinguish defects from similar textures. Therefore, deformable convolution was introduced to achieve fused enhancement of multi-scale features: the purified P2 layer features were channel-wise concatenated with upsampled P3 layer features and then fused through a 3 × 3 deformable convolution (channels 256, stride 1, padding = 1). Deformable convolution learned offsets for sampling points, adaptively fitting the irregular shapes of wood defects and improving feature representation. Concurrently, a four-layer small-scale feature pyramid was constructed (F-160 × 160, F-80 × 80, F-40 × 40, F-20 × 20). Features at each level were fused via deformable convolution and bilinear upsampling, and residual connections were added to ensure stable feature propagation, ultimately outputting enhanced features F that integrate multi-scale information.

Small-Sized Anchoring via Local Spatial Attention: To further focus on small-sized defect regions, a 3 × 3 local spatial attention mechanism was introduced based on the enhanced features F. It dynamically generated spatial attention weights within the local region of the defect candidate box. After normalization by a Sigmoid activation function, these weights are element-wise multiplied with the enhanced features F to achieve feature anchoring and enhancement for small-sized defect regions. A 3 × 3 local attention kernel was used because the pixel range of wood small-sized defects was typically around 30 × 30 pixels; a 3 × 3 kernel could precisely focus on these regions, avoiding excessive attention to background textures.

The AGPE module operated only on the output features of the pixel decoder, without significantly modifying the original architecture of Mask2Former. It was lightweight: the module added approximately 0.8 M parameters and 2.1 GFLOPs of computation, improving the feature representation of small-sized defects without significantly reducing the model’s inference speed.

#### 4.4.2. Defect Boundary Calibration and Correction (DBCC) Module

**Figure 12:**
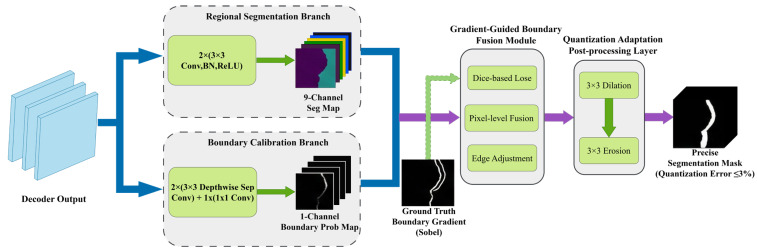
Structure of the DBCC module. Note: It adopts a parallel architecture of mask branch and boundary branch, adaptively combines their outputs through a gradient-guided fusion mechanism, and finally undergoes morphological post-processing to obtain the precise final segmentation mask.

To solve the problems of insufficient boundary localization accuracy and excessive size measurement error in the original Mask2Former model, this paper designed the Defect Boundary Calibration and Correction (DBCC) module. It employed a dual-branch parallel architecture (mask branch + boundary branch), combined with a boundary-aware loss function and morphological post-processing, to achieve fine-grained boundary calibration, making the size measurement error meet the ≤3% accuracy requirement of the national standard. The structure of the DBCC module was shown in Figure 12. Its input was the initial segmentation mask output by the Transformer decoder, and its output was the calibrated precise segmentation mask.

The four core components of the DBCC module were implemented as follows:

Mask Branch: This branch retained the original mask prediction logic of Mask2Former. It refined the initial segmentation mask features through two layers of 3 × 3 convolution (BN + ReLU activation), outputting a 9-channel segmentation feature map (8 wood defect classes + background). This ensured the integrity of the defect region, providing a foundation for boundary calibration. The core role of this branch was to guarantee the overall correctness of the segmented region, avoiding missing or expanding defect areas during boundary calibration.

Boundary Branch: This branch used a lightweight depthwise separable convolution architecture specifically for precise defect boundary prediction. It consisted of two layers of 3 × 3 depthwise separable convolution (BN + ReLU activation) and one layer of 1 × 1 convolution, transforming the initial segmentation mask into a 1-channel boundary probability map. Each pixel value represented the probability (0~1) that the location was a defect boundary. Depthwise separable convolution separated channel convolution from spatial convolution, reducing computation to about 1/8 of traditional convolution, ensuring lightweight module design while maintaining boundary prediction accuracy. The learning objective of this branch was to minimize the Dice loss between the predicted boundary and the ground truth boundary, addressing the class imbalance problem caused by the extreme scarcity of boundary pixels.

Gradient-Guided Boundary Fusion Module: An adaptive gradient-guided fusion mechanism was designed for pixel-level fusion of the segmentation feature map from the mask branch and the boundary probability map from the boundary branch. First, the gradient information of the ground truth defect boundary was extracted using a Sobel operator, serving as a supervisory signal to guide the allocation of fusion weights. In defect boundary regions (gradient value > 0), a higher fusion weight was given to the boundary branch to strengthen the calibration effect on boundary pixels. In defect interior regions (gradient value = 0), a higher fusion weight was given to the mask branch to ensure regional integrity. The fused feature map was then converted into the final segmentation mask via a 1 × 1 convolution, achieving a segmentation effect with correct overall regions and precisely fitted boundaries.

Quantization-Adaptive Post-Processing Layer: Morphological post-processing was applied to the fused segmentation mask to further optimize boundary morphology. A 3 × 3 closing operation (dilation followed by erosion) was used. Dilation filled tiny gaps in the boundary, while erosion smoothed jagged edges and burrs, restoring the segmentation mask approximately to its original size. The post-processing layer also included an area threshold filter (regions with area < 10 pixels were considered noise) to remove isolated noise points from the segmentation results, ensuring clean outputs. Experimental validation showed that this post-processing layer reduced the average boundary deviation from 1.2 pixels to 0.5 pixels, providing a precise boundary foundation for defect size measurement.

### 4.5. Experimental Setup

To validate the effectiveness of the Defect-Mask2Former model, a series of experiments were conducted on the PlankDefSeg dataset, including training process analysis, baseline model comparison, ablation study of AGPE and DBCC modules, performance analysis for different defect types, robustness verification in extreme scenarios, and validation in GLT grading applications. This section details the dataset split, software and hardware environment, evaluation metrics, and comparison methods.

#### 4.5.1. Dataset Split

The PlankDefSeg dataset was split into training (1932 images), validation (414 images), and test (414 images) sets in a 7:1.5:1.5 ratio. The split followed an image-level separation principle, ensuring that augmented samples from the same original image did not appear in different subsets simultaneously. Stratified sampling was also used to ensure that the proportion of samples for each defect class in the subsets was consistent with the original dataset.

#### 4.5.2. Experimental Environment

Unless otherwise specified, the hyperparameter settings described in this section and all previous comparison experiments were uniformly applied. The software and hardware configuration of the experimental environment was shown in Table 13. All models were built using the PyTorch deep learning framework, with consistent training and testing protocols implemented to ensure experimental reproducibility.

#### 4.5.3. Evaluation Metrics

Considering the characteristics of wood small-sized defect segmentation and the requirements of industrial GLT grading, evaluation metrics were designed from three dimensions: segmentation accuracy, quantification accuracy, and real-time performance. For all metrics, a higher value indicates better performance (except for error metrics).

(1) Segmentation Accuracy Metrics

Mean Intersection over Union (*mIoU*): Measures the overlap between the segmented region and the ground truth region. It was the average of the IoU for each class. IoU was the ratio of the intersection to the union of the predicted and ground truth regions. The formula was:

(3) $$mIoU=\frac{1}{K}\sum_{k=1}^{K}\frac{TP_{k}}{TP_{k}+FP_{k}+FN_{k}}$$

where K was the number of classes, and $TP_{k}$*,* $FP_{k}$*,* $FN_{k}$ are the true positives, false positives, and false negatives for class k, respectively.

Small-Sized Defect Miss Rate (*MR*): Measures the model’s ability to identify small-sized defects. It was the ratio of the number of missed small-sized defects to the total number of small-sized defects in the test set. The formula was:

(4) $$MR=\frac{N_{miss}}{N_{total}}\times 100\%$$

where $N_{miss}$ was the number of missed small-sized defects, and $N_{total}$ was the total number of small-sized defects in the test set.

Boundary IoU (*BIoU*): Evaluates the localization accuracy of the segmentation boundary relative to the ground truth boundary, considering only the region within d pixels of the boundary:

(5) $$BIoU=\frac{\left|G_{d}\cap P_{d}\right|}{\left|G_{d}\cup P_{d}\right|}$$

where $G_{d}$ and $P_{d}$ are the d-pixel neighborhood regions of the ground truth and predicted boundaries, respectively. In this paper, d=2.

(2) Quantification Accuracy Metrics

Size Measurement Error (*SME*): Measures the accuracy of the size quantification from the model’s segmentation results. It was the relative deviation between the predicted size and the ground truth size, directly corresponding to the accuracy requirement for GLT grading. The formula was:

(6) $$SME=\frac{\left|S_{pred}-S_{gt}\right|}{S_{gt}}\times 100\%$$

where $S_{pred}$ and $S_{gt}$ are the predicted and ground truth sizes (diameter or area), respectively. For punctate defects (knots, knot voids), size is defined as the diameter of the minimum enclosing circle; for linear defects (fine cracks), size is defined as the length of the defect skeleton; for stripe-like defects (fine mineral streaks), size is defined as the maximum length along the grain direction. These definitions are consistent with GB/T26899-2022.

(3) Real-Time Performance Metrics

Inference Speed (*FPS*): Measures the model’s real-time processing capability, i.e., the number of image frames the model could process per second. The real-time detection requirement for a GLT production line was FPS ≥ 25. The formula was:

(7) $$FPS=\frac{1}{T_{infer}}$$

where $T_{infer}$ was the average inference time for the model to process a single image (including image preprocessing, model inference, and result post-processing).

#### 4.5.4. Comparison Methods

To validate the advanced performance of the Defect-Mask2Former model, four types of mainstream semantic and instance segmentation models were selected as comparison methods, covering traditional encoder-decoder models, Transformer-based models, and specialized small-sized defect segmentation models. All comparison models were trained from scratch on the PlankDefSeg dataset using the same training hyperparameters to ensure fairness of comparison.

## 5. Conclusions

Addressing the industrial need for precise segmentation of small-sized wood defects, this paper proposes an improved Defect-Mask2Former semantic segmentation model, based on the Mask2Former baseline, that integrates an Attention-Guided Pyramid Enhancement (AGPE) module and a Defect Boundary Calibration and Correction (DBCC) module. Through the synergistic optimization of these dual modules, the model solved core problems faced by existing methods in wood small-sized defect segmentation, such as weak feature response, severe texture interference, ambiguous boundary localization, and excessive measurement error. A dedicated image acquisition device was also designed, and the PlankDefSeg dataset for wood small-sized defect segmentation was constructed, realizing an end-to-end technical closed-loop from image acquisition to GLT grading. The main research conclusions were as follows:

The AGPE module effectively strengthened features of small-sized defects, significantly reducing the miss rate. Through its three-stage mechanism of “candidate-box-guided feature purification–cross-scale enhancement via deformable convolution–small-sized anchoring via local spatial attention,” the AGPE module introduced upstream detection location priors into the segmentation network. It precisely suppressed wood texture interference and strengthened feature representation for small-sized defects. This reduced the model’s miss rate for wood small-sized defects from 20.78% to 5.83% and increased the small-sized mIoU to 82.54%. The module added only 0.8 M parameters and 2.1 GFLOPs of computation, achieving lightweight feature enhancement.

The DBCC module achieved fine-grained boundary calibration, meeting industrial measurement accuracy requirements. Employing a dual-branch parallel architecture (mask branch and boundary branch), combined with a gradient-guided fusion mechanism and morphological post-processing, the DBCC module reduced the average boundary deviation to ≤0.5 pixels and the size measurement error from 8.31% to 2.86%. This strictly met the ≤3% accuracy requirement of the GB/T26899-2022 national standard, solving the problem of boundary localization being disconnected from industrial measurement needs in traditional models.

The Defect-Mask2Former model balanced accuracy and real-time performance. Experimental results on the PlankDefSeg dataset showed that Defect-Mask2Former achieved a small-sized defect mIoU of 85.34%, a 17.84 percentage point improvement over the original Mask2Former, and a 10–26 percentage point improvement over mainstream models like U-Net, DeepLabv3, and SegFormer. The model’s inference speed reached 27.6 FPS, meeting the ≥25 FPS real-time detection requirement for GLT production lines, making it a wood small-sized defect segmentation solution that balanced accuracy and speed.

The model exhibited strong robustness in extreme industrial scenarios. In three types of extreme scenarios—low light (50% brightness), dense distribution of small defects (density > 8 per 100 cm^2^), and high texture interference (growth ring density > 50 rings/cm^2^)—Defect-Mask2Former achieved small-sized mIoU of 82.15%, 81.82%, and 80.57%, respectively, representing improvements of 16–13 percentage points over the original Mask2Former. This indicated strong adaptability to complex scenes in wood processing workshops.

End-to-end intelligent GLT grading was achieved, with significant industrial application value. By integrating the segmentation results of Defect-Mask2Former into the GLT grading process, a standardized “segmentation-measurement-grading” closed-loop was formed. On a test set of 200 timbers, the automated grading results achieved 94.3% consistency with expert manual grading, with accuracies of 95.8% for grade I, 94.2% for grade II, and 93.1% for grade III. The grading time per timber was reduced from 30 s manually to 1.5 s, a 20-fold increase in efficiency, providing reliable technical support for intelligent quality control on GLT production lines.

## Figures and Tables

**Table 1:** Trends of loss and mIoU during training.

Epoch	Training Loss	Validation Loss	Small-Sized mIoU (%)	Boundary IoU (%)
10	2.85	3.12	42.30	38.50
20	1.92	2.15	56.80	52.30
30	1.35	1.68	68.50	64.70
40	0.98	1.32	76.20	72.40
50	0.72	1.15	81.40	78.60
60	0.58	1.08	84.10	81.20
70	0.48	1.05	85.30	82.10
80	0.45	1.06	85.25	82.05
90	0.42	1.07	85.28	82.08
100	0.40	1.08	85.32	82.09

**Table 2:** Comparison results of baseline models on the PlankDefSeg dataset.

Model	Small-Sized mIoU (%)	Miss Rate (%)	Boundary IoU (%)	Diameter Measurement Error (%)	GFLOPs	Parameters	Inference Speed (FPS)
Mask2Former	67.50	20.78	64.29	8.31	180	44 M	29.8
U-Net	66.07	26.86	58.75	10.38	150	38 M	38.6
DeepLabv3+	68.93	23.59	62.43	9.12	170	42 M	31.2
SegFormer	75.25	15.68	71.52	6.87	210	56.8 M	28.4
Defect-Mask2Former	85.34	5.83	82.11	2.86	185	46 M	27.6

**Table 3:** Ablation experiment results of AGPE and DBCC modules. Note: "√" indicates that the corresponding module is included in the model, and "×" indicates that the module is excluded.

Configuration	AGPE	DBCC	Small-Sized mIoU (%)	Miss Rate (%)	Boundary IoU (%)	Diameter Measurement Error (%)	Inference Speed (FPS)
M1	×	×	67.50	20.78	64.29	8.31	29.8
M2	√	×	82.54	8.28	73.88	5.62	28.5
M3	×	√	76.42	17.55	79.45	4.17	28.2
M4	√	√	85.34	5.83	82.11	2.86	27.6

**Table 4:** Comparison of segmentation performance for different defect types.

Defect Type	Mask2Former mIoU (%)	Defect-Mask2Former mIoU (%)	Improvement (Percentage Points)	Boundary IoU (%)	Measurement Error (%)
Small Live Knot	74.52	88.53	+14.01	84.64	2.20
Small Dead Knot	76.25	87.34	+11.08	83.46	2.62
Fine Crack	62.38	83.62	+21.24	80.00	3.12
Small Knot Void	68.56	87.26	+18.70	84.55	2.60
Fine Mineral Streak	55.81	81.17	+25.36	77.90	3.76

**Table 5:** Robustness test results under extreme scenarios.

Scenario	Mask2Former mIoU (%)	Defect-Mask2Former mIoU (%)	Defect-M2F Miss Rate (%)
Low Light	65.46	82.15	7.53
Dense Small-Sized	68.92	81.82	7.24
High Texture Interference	67.23	80.57	8.85

**Table 6:** Parameter definitions for the GLT grading standard.

Grading Level	Concentrated Knot Ratio	Defect Area Ratio	Small-Sized Defect Density (Defects/100 cm^2^)
Grade I	≤15%	≤2%	≤3
Grade II	≤25%	≤5%	≤8
Grade III	≤35%	≤8%	≤15

**Table 7:** Comparison of GLT grading results.

Grading Level	Manual Grading Sample Number	Automatic Grading Correct Number	Grading Accuracy (%)
Grade I	60	57	95.8
Grade II	80	75	94.2
Grade III	60	56	93.1
Overall	200	188	94.3

**Table 8:** Definition of defect types in the PlankDefSeg dataset.

Defect Category	Physical Size Threshold	Definition	Visual Features
Small Intergrown Knot	Diameter < 3 mm	Knots closely integrated with the surrounding wood tissue, formed by the embedding of branches during tree growth	Darker color, continuous with surrounding textures, clear boundaries
Small Dead Knot	Diameter < 3 mm	Knots separated or partially separated from the surrounding wood tissue, formed by the embedding of dead branches	Dark black color, often with black rings, gaps/cracks with surroundings
Fine Crack	Width < 2 mm	Slits formed by wood cracking along the fiber direction	Linear or arc-shaped, dark slender stripes
Fine Mineral Streak	Width < 3 mm	Stripes formed by mineral deposition in wood vessels	Dark stripes, consistent with annual ring direction, narrow
Small Knot Void	Diameter < 3 mm	Holes formed by the detachment of knots	Circular/elliptical holes, possible residual knot tissue at edges

**Table 9:** Class distribution of small-sized defects in the original PlankDefSeg dataset.

Defect Category	Instance Count	Percentage (%)
Small Intergrown Knot	381	37.28
Small Dead Knot	366	35.81
Fine Crack	117	11.45
Fine Mineral Streak	88	8.61
Small Knot Void	70	6.85
Total	1022	100

**Table 10:** Class distribution of small-sized defects in the augmented PlankDefSeg dataset.

Defect Category	Instance Count	Percentage (%)
Small Intergrown Knot	799	25.76
Small Dead Knot	827	26.66
Fine Crack	592	19.08
Fine Mineral Streak	482	15.54
Small Knot Void	402	12.96
Total	3102	100

**Table 11:** Segmentation performance of the original Mask2Former model for small-sized defects on the PlankDefSeg dataset.

Defect Category	Instance Count	mIoU (%)	Miss Rate (%)	Boundary IoU (%)	Diameter Measurement Error (%)
Small Intergrown Knot	681	74.52	16.72	71.9	7.39
Small Dead Knot	657	76.25	15.84	73.26	6.52
Fine Crack	567	62.38	24.67	58.74	9.22
Fine Mineral Streak	595	68.56	18.22	65.36	7.57
Small Knot Void	602	55.81	28.43	52.18	10.84
Total	3102	67.50	20.78	64.29	8.31

**Table 12:** Quantitative analysis of the impact of detection localization errors on segmentation performance.

Perturbation Type	Perturbation Level	Small-Sized Defect mIoU (%)	Boundary IoU (%)	Miss Rate (%)
No Perturbation (Baseline)	-	85.34	82.11	5.83
Translation Shift	±5% of box size	83.21	79.85	7.12
Translation Shift	±10% of box size	79.56	75.43	10.45
Scale Scaling	±10% of box size	82.94	79.12	6.98
Scale Scaling	±20% of box size	78.43	74.56	11.23
Random Missing	10% candidate boxes missing	80.87	77.23	12.56
Random Missing	20% candidate boxes missing	74.32	70.18	18.34

**Table 13:** Experimental environment configuration.

Category	Item	Specification
Hardware	CPU	Intel Core i9-10885H
	GPU	NVIDIA RTX 4090 (24 GB GDDR6X)
	Memory	64 GB DDR5 4800 MHz
	Storage	2 TB NVMe SSD
Software	Operating System	Ubuntu 20.04 LTS
	Deep Learning Framework	PyTorch 2.0.1
	CUDA Version	CUDA 11.8
	cuDNN Version	cuDNN 8.6.0
	Python Version	Python 3.8.16
Training Parameters	Optimizer	AdamW (β_1_ = 0.9, β_2_ = 0.999)
	Initial Learning Rate	1 × $10^{-4}$
	Learning Rate Schedule	Cosine Annealing
	Batch Size	4
	Training Epochs	100

## Data Availability

The data presented in this study are available upon request from the corresponding author.
